# MAPLE Coatings Embedded with Essential Oil-Conjugated Magnetite for Anti-Biofilm Applications

**DOI:** 10.3390/ma14071612

**Published:** 2021-03-25

**Authors:** Oana Gherasim, Roxana Cristina Popescu, Valentina Grumezescu, George Dan Mogoșanu, Laurențiu Mogoantă, Florin Iordache, Alina Maria Holban, Bogdan Ștefan Vasile, Alexandra Cătălina Bîrcă, Ovidiu-Cristian Oprea, Alexandru Mihai Grumezescu, Ecaterina Andronescu

**Affiliations:** 1 Department of Science and Engineering of Oxide Materials and Nanomaterials, Faculty of Applied Chemistry and Materials Science, Politehnica University of Bucharest, 1-7 Gheorghe Polizu Street, 011061 Bucharest, Romania; oana.gherasim@inflpr.ro (O.G.); bogdan.vasile@upb.ro (B.Ș.V.); ada_birca@yahoo.com (A.C.B.); grumezescu@yahoo.com (A.M.G.); ecaterina.andronescu@upb.ro (E.A.); 2 Lasers Department, National Institute for Lasers, Plasma and Radiation Physics, 409 Atomistilor Street, 077125 Magurele, Romania; 3 Department of Life and Environmental Physics, “Horia Hulubei” National Institute for Physics and Nuclear Engineering, 30 Reactorului Street, 077125 Magurele, Romania; roxana.popescu@nipne.ro; 4 Department of Pharmacognosy and Phytotherapy, Faculty of Pharmacy, University of Medicine and Pharmacy of Craiova, 2 Petru Rares Street, 200349 Craiova, Romania; mogosanu2006@yahoo.com; 5 Research Center for Microscopic Morphology and Immunology, University of Medicine and Pharmacy of Craiova, 2 Petru Rares Street, 200349 Craiova, Romania; laurentiu_mogoanta@yahoo.com; 6 Department of Biochemistry, Faculty of Veterinary Medicine, University of Agronomic Science and Veterinary Medicine, 59 Marasti Boulevard, 011464 Bucharest, Romania; floriniordache84@yahoo.com; 7 Department of Microbiology and Immunology, Faculty of Biology, University of Bucharest, 91-95 Splaiul Independentei Street, 077206 Bucharest, Romania; alina_m_h@yahoo.com; 8 Research Institute of the University of Bucharest—ICUB, University of Bucharest, 90-92 Panduri Road, 050657 Bucharest, Romania; 9 Department of Inorganic Chemistry, Physical Chemistry and Electrochemistry, Faculty of Applied Chemistry and Materials Science, Politehnica University of Bucharest, 1-7 Gheorghe Polizu Street, 011061 Bucharest, Romania; ovidiu.oprea@upb.ro

**Keywords:** magnetite, essential oil, MAPLE, composite coatings, anti-biofilm efficiency

## Abstract

The present study reports on the development and evaluation of nanostructured composite coatings of polylactic acid (PLA) embedded with iron oxide nanoparticles (Fe_3_O_4_) modified with Eucalyptus (*Eucalyptus globulus*) essential oil. The co-precipitation method was employed to synthesize the magnetite particles conjugated with Eucalyptus natural antibiotic (Fe_3_O_4_@EG), while their composition and microstructure were investigated using grazing incidence X-ray diffraction (GIXRD), Fourier transform infrared spectroscopy (FT-IR), thermogravimetric analysis (TGA), transmission electron microscopy (TEM) and dynamic light scattering (DLS). The matrix-assisted pulsed laser evaporation (MAPLE) technique was further employed to obtain PLA/Fe_3_O_4_@EG thin films. Optimal experimental conditions for laser processing were established by complementary infrared microscopy (IRM) and scanning electron microscopy (SEM) investigations. The in vitro biocompatibility with eukaryote cells was proven using mesenchymal stem cells, while the anti-biofilm efficiency of composite PLA/Fe_3_O_4_@EG coatings was assessed against Gram-negative and Gram-positive pathogens.

## 1. Introduction

Traditional medicine relies on exploring the experience-based therapeutic benefits of extracts and infusions from different parts of plants and herbs. However, this rather temporary and non-selective approach and the current advances in pharmaceutical sciences determined an increased interest for new and performance-enhanced formulations. The intrinsic medicinal properties of phytochemicals have been acknowledged for a long period of time [1,2], but the interest for such natural-derived bioactive substances has lately grown considerably [3,4,5,6,7,8,9,10].

Given the actual context of alarming increasing prevalence of antibiotic-resistant bacteria [11,12], particular attention was oriented on developing new antimicrobial formulations. For example, extracts of acacia gum [13,14], cinnamon [15,16], clove [17,18], cumin [19,20], lavender [21,22], oregano [23,24], rosemary [25,26], sage [27,28], tea tree [29,30] and thyme [31,32] were reported as promising alternatives to combat the growth of planktonic microorganisms, as well as the formation and development of bacterial biofilms.

Substantial antibacterial effects of Eucalyptus essential oils were evidenced against *Escherichia coli* and *Staphylococcus aureus* by Bachir & Benali, with a more prominent effect being reported on the Gram-negative strain [33]. The increased efficiency of essential oils derived from different Eucalyptus species against Gram-negative pathogens [34,35] was mainly related to the abundance of a water-insoluble 1,8–cineole (eucalyptol) component, which may cause the disruption of bacterial outer membrane and the leakage of cytoplasm [36,37]. Complementary studies evaluated the correlation between the chemical composition and the antibacterial efficiency of essential oils from different Eucalyptus species, proving their potential application in pharmaceutical products [38,39].

To reach the required therapeutic concentrations, high doses from traditional pharmaceutical formulations are usually administered. Modern drug delivery systems represent an attractive, versatile and effective approach to overcome the limitations of regular medicines, such as: (i) side effects; (ii) poor drug concentration at the targeted place; (iii) the rapid metabolizing rate; and (iv) the degradation of active substances.

By gathering the great outcome of nanotechnology and the therapeutic implications of plant-derived compounds, new pharmaceutical formulations were lately developed and thoroughly evaluated. Therefore, an impressive extension of the medicinal use of phytochemicals was reported, including unconventional antioxidants [40,41,42,43,44] and antimicrobials [45,46,47,48], wound dressings [49,50,51], dermal products [52,53,54,55] and anti-cancer platforms [56,57,58,59].

A particular application of nanotechnology-derived unconventional formulations consists of optimizing the surface of commonly used medical devices with nanostructured and bioactive coatings, intending to potentiate or induce anti-infective properties [60,61,62]. The development of antimicrobial and anti-biofilm coatings that combat drug-resistant microorganisms and reduce or eliminate related infections is of great interest for the healthcare system.

Magnetite (Fe_3_O_4_) is the most explored representative of iron oxides, with remarkable implications for biomedical use [63,64,65]. Besides facile and significant yield synthesis, Fe_3_O_4_ particles possess peculiar nanosize-related features, such as surface functionalization, tunable biocompatibility and superparamagnetic behavior [66,67,68,69,70]. The surface modification of magnetite nanoparticles with essential oils represents a bidirectional strategy to obtain multifunctional platforms: (i) firstly, the organic molecules act as modulators for the compatibility and stability of Fe_3_O_4_ nanoparticles; and (ii) secondly, the inorganic core acts as a stabilizer and potentiating agent for the essential oils [71,72]. By combining these benefits, new and effective therapeutic nanosystems can be provided, with the additional ability of targeted and controlled treatment.

For this reason, we report herein the deposition of composite films based on polylactic acid (PLA) embedded with magnetite nanoparticles in situ conjugated with Eucalyptus essential oil (Fe_3_O_4_@EG). The chemical co-precipitation method employed in the study offers a high yield reaction and reproducibility in the synthesis of Eucalyptus-conjugated nanoparticles. The embedding of Fe_3_O_4_@EG systems within polymeric films of PLA was done to protect the active substance, improve the system’s biocompatibility, implement a slow release of the active substance, and protect the considered medical device of eventual oxidation and degradation processes. Given the promising data on the matrix-assisted pulsed laser evaporation (MAPLE) method towards the fabrication of bioactive, stoichiometric and homogenous coatings [73,74], this technique was used in order to obtain nanostructured PLA/Fe_3_O_4_@EG surfaces.

## 2. Materials and Methods

### 2.1. Materials

All reagents used to synthesize nanosystems, and nanostructured coatings were acquired from Sigma-Aldrich (Merck Group, Darmstadt, Germany), if not specified. All chemicals were characterized by analytical purity, according to the American Chemical Society (ACS), and all solutions were prepared using deionized water (MiliQ^®^, Merck Millipore, Burlington, MA, SUA).

*Eucalyptus globulus* (EG) essential oil and 1 cm^2^ polished (1 0 0) silicon (Si) and glass substrates were provided by a local supplier.

Cell cultures, i.e., human mesenchymal stem cells derived from amniotic fluid and bacterial strains of *Escherichia coli* (ATCC**^®^** 15224) and *Staphylococcus aureus* (ATCC**^®^** 25923), were obtained from the American Type Culture Collection (ATCC, Manassas, VA, USA).

### 2.2. Methods

#### 2.2.1. Synthesis of Fe_3_O_4_@EG Nanoparticles

Magnetite nanoparticles conjugated with Eucalyptus essential oil (Fe_3_O_4_@EG) were obtained using a modified chemical co-precipitation method, in compliance with previous studies [75,76]. In this respect, metallic precursor solution was obtained by dissolving ferric chloride (FeCl_3_) and ferrous sulfate (FeSO_4_∙7H_2_O) in MiliQ. The resulted solution was subsequently added drop by drop within an alkaline solution consisting of 25% ammonium hydroxide (NH_3_∙OH), EG essential oil and MiliQ. The process was completed under magnetic stirring, and the resulted precipitate was washed several times with deionized water under magnetic separation. The final product was dried at 40 °C for 6 h, under an inert atmosphere.

#### 2.2.2. Synthesis of PLA/Fe_3_O_4_@EG Coatings

Before MAPLE processing, all substrates were subsequently cleaned for 30 min with acetone, ethanol and MiliQ in an ultrasonic bath, then dried under a high-purity nitrogen jet. The solid MAPLE targets were prepared by freezing at liquid nitrogen temperature the suspension obtained by mixing poly(D,L-lactide) (PLA) and Fe_3_O_4_@EG in dimethyl sulfoxide (DMSO).

The as-obtained frozen targets were further irradiated with a KrF^*^ excimer laser beam (λ = 248 nm, τ_FWHM_ = 25 ns), using a COMPexPro 205 Lambda Physics source from Coherent (Göttingen, Germany). During MAPLE processing, experimental parameters were set as follows: room temperature and 0.1 Pa pressure inside the deposition chamber, 4 cm target-to-substrate distance, 0.4 Hz and 15 Hz target rotation and laser repetition frequency, respectively. 30,000 laser pulses were applied at different fluences (200, 300 and 400 mJ/cm^2^) to obtain PLA/Fe_3_O_4_@EG coatings.

#### 2.2.3. Physiochemical Characterization

##### X-Ray Diffraction (XRD)

To investigate the purity and crystallinity of synthesized powdery sample, grazing incidence XRD analysis was performed using a PANalytical Empyrean diffractometer (Almelo, the Netherlands) with Cu_Kα_ radiation (λ = 1.541874 Å), equipped with a 2 × GE (2 2 0) hybrid monochromator for Cu and a parallel plate collimator on the PIXcel3D detector. The scanning was done for 2θ diffraction angles in the 20–80° range, with an incidence angle of 0.5°, a step dimension of 0.04° and a time step of 3 s.

##### Fourier Transform Infrared Spectroscopy (FT-IR)

Compositional aspects on the synthesized powder were obtained using a Nicolet 6700 FT-IR spectrometer (Thermo Fischer Scientific, Waltham, MA, USA) connected to the OmnicPicta 8.2 software. To get the FT-IR spectrum, 32 scans were recorded in the 4000–400 cm^−1^ frequency range, with 4 cm^−1^ resolution. Scanning was done under a controlled ambient temperature in the attenuated total reflectance mode.

##### Thermogravimetric Analysis (TGA)

The thermal behavior of pristine and EG-conjugated iron oxide powders was investigated using Shimadzu DTG-TA-50H equipment (Carlsbad, CA, USA). Small amounts of powdery samples were placed in alumina crucibles, heated from room temperature to 1000 °C with a heating rate of 1 °C/min, in a normal atmosphere.

##### Transmission Electron Microscopy (TEM)

Relevant microstructural data on the synthesized particles were provided by TEM analysis, which was performed with a Tecnai^TM^ G2 F30 S-TWIN instrument equipped with a selected area electron diffraction (SAED) accessory (FEI Company, Thermo Fisher Scientific, Hillsboro, OR, USA). The microscope was used in the transmission mode, at a 300 kV voltage, with point and line resolutions of 2 Å and 1 Å, respectively. For analysis, serial ethanol dilutions were obtained, and the final suspension was placed on a holey carbon-copper grid for investigation.

##### Dynamic Light Scattering (DLS)

The hydrodynamic diameter and zeta potential of EG-conjugated particles were de-termined using a DelsaMax Pro instrument from Beckman Coulter (Brea, CA, USA). Prior to the analysis (performed with a 532 nm laser beam), ultrapure water dilution was pre-pared by ultrasound dispersion at room temperature.

##### Infrared Microscopy (IRM)

Relevant compositional information on the MAPLE processed materials was pro-vided by IRM analysis, which was made using a Nicolet iN10 MX FT-IR microscope (Thermo Fischer Scientific Company, Waltham, MA, USA). Measurements were recorded between 4000 and 700 cm^−1^ at 4 cm^−1^ resolution, in the reflection mode. Thirty-two scans were collected for each sample, then co-added and converted to absorbance using the OmincPicta 8.0 software (Thermo Fischer Scientific).

##### Scanning Electron Microscopy (SEM)

A FEI Quanta Inspect F scanning electron microscope (Thermo Fisher Scientific, Hillsboro, OR, USA) was used to evaluate the morphology of PLA/Fe_3_O_4_@EG films. Prior to analysis, samples were capped with a thin gold layer and the SEM micrographs were recorded using secondary electron beams (energy of 30 keV). For cross-section investigation, the (1 0 0) Si substrates used during MAPLE processing were cut with a diamond disc.

#### 2.2.4. In Vivo Biodistribution of Fe_3_O_4_@EG Nanoparticles

To evaluate the in vivo effects of Fe_3_O_4_@EG nanosystems, suspensions were prepared in phosphate-buffered saline (1 mg/mL concentration) and subjected to UV treatment (30 min). Volumes of 100 μL from as-obtained sterile suspensions were aseptically administered into the left jugular vein of three-weeks-old BALB/c mice, under general anesthesia (Ketamine/Xylazine mixture). Reference mice were injected with 100 μL of phosphate-buffered saline (PBS). After inoculation, all animals were kept in standard conditions and received food and water *ad libitum*.

After 2 days and 10 days of treatment, animals were euthanized under general anesthesia, and internal organs (brain, myocardium, liver, lung, pancreas, kidney and spleen) were harvested. The harvested organs were washed with PBS, preserved in 10% buffered neutral formalin (72 h, room temperature) and prepared for paraffin processing. Serial cross-sections of 4 μm thickness were cut using an HM355s rotary microtome equipped with a waterfall-based section transfer system (MICROM International GmbH, Walldorf, Germany). The as-obtained tissue fragments were placed on histological slides, treated with poly-L-Lysine and the classical Hematoxylin–Eosin (HE) staining protocol was applied. Cross-section tissue samples were examined and photographed with a Nikon Eclipse 55i light microscope coupled with a Nikon DS–Fi1 CCD high definition video camera from Nikon Instruments (Apidrag, Bucharest, Romania). Optical micrographs were captured, stored and processed using the Image-Pro Plus 7.0.0 software from Media Cybernetics Inc. (Buckinghamshire, UK).

Animal experiments within this study were performed in compliance with all European Commission directives on animal experiments and were approved by the Ethics Committee of the University of Medicine and Pharmacy of Craiova, Romania.

#### 2.2.5. In Vitro Evaluation of PLA/Fe_3_O_4_@EG Coatings

##### Biocompatibility Evaluation

The biocompatibility of composite materials was quantitatively assessed on amniotic fluid-derived mesenchymal stem cells (AFSCs) using the MTT tetrazole (3-(4,5-dimethylthiazol-2-yl)-2,5-diphenyltetrazolium bromide) viability assay. For biological evaluation, reduced amounts of PLA/Fe_3_O_4_@EG were obtained by scratching an area of 0.25 cm^2^ from the MAPLE-coated glass substrates. All samples were sterilized using UV irradiation.

AFSCs were seeded at 3000 cells/well cellular densities in 100 µl of Dulbecco’s Modified Eagle Medium supplemented with 10% fetal bovine serum and 1% antibiotic mixture (Penicillin/Streptomycin/Neomycin), using 96-well plates (Thermo Fisher Scientific, Waltham, MA, USA). After 72h of standard incubation (37 ± 2 °C, 5 ± 1% CO_2_, humid atmosphere), the viability of cells was quantitatively determined using the Vybrant**^®^** MTT Cell Proliferation Assay Kit (Thermo Fischer Scientific). Two supplementary dark incubation periods (4h each) were required after the addition of 10 μL of 12 mM MTT solution and 100 μL of SDS-HCl solubilizing solution in each well, respectively. The absorbance values for each specimen were spectrophotometrically measured at 570 nm, using in this respect a Mithras LB 940 instrument (Berthold Technology, Bad Wildbad, Germany). The MTT viability assay was performed in triplicate experiments at different time intervals.

##### Anti-Adherent Potential

The ability of PLA/Fe_3_O_4_@EG materials to interfere with the development of bacterial biofilms of *Escherichia coli* (*E. coli*) and *Staphylococcus aureus* (*S. aureus*) was experimentally evaluated. All samples (uncoated and MAPLE modified glass substrates) were sterilized using 20 min of UV irradiation. Each sample was individually placed in wells of a 6-well plate (Nunc), followed by the addition of 2 mL of Luria-Bertani (LB) broth and subsequent inoculation of 50 μL of microbial suspensions (0.5 McFarland standard densities). After 24h of incubation in standard conditions, the samples were washed with sterile physiological PBS, the culture medium was removed and fresh LB medium was added to ensure the growth of microbial biofilm. After 24, 48 and 72 h of incubation, specimens were washed with PBS and placed into PBS-containing sterile tubes for subsequent vortexing (30 s). The biofilm-forming cell suspensions were diluted and seeded onto solidified LB agar plates in order to evaluate the colony-forming units (CFU/mL). All experiments were performed in triplicate.

## 3. Results & Discussions

For the synthesis of conjugated iron oxide nanoparticles, we used a modified chemical co-precipitation method, which implied using Fe^2+^:Fe^3+^ in a 1.6:1 ratio. The contribution of trivalent cation was smaller to compensate the oxidation of Fe^2+^ [77,78], while the precipitation condition was provided by properly adjusting the pH value around 11 with a weak alkaline solution. The organic phase (Eucalyptus essential oil) was introduced in the precipitation medium to form micelles, limiting the nucleation stage of nanoparticles and ensuring a better control of their growth and dimension [79,80]. The molecules of EG essential oil also acted as an in situ functionalizing agent by establishing weak interactions with the abundant hydroxyl groups on the surface of nanoparticles [81,82]. The synthesized iron oxide powder was subjected to characterization by means of composition, crystallinity and morphology.

**Figure 1:**
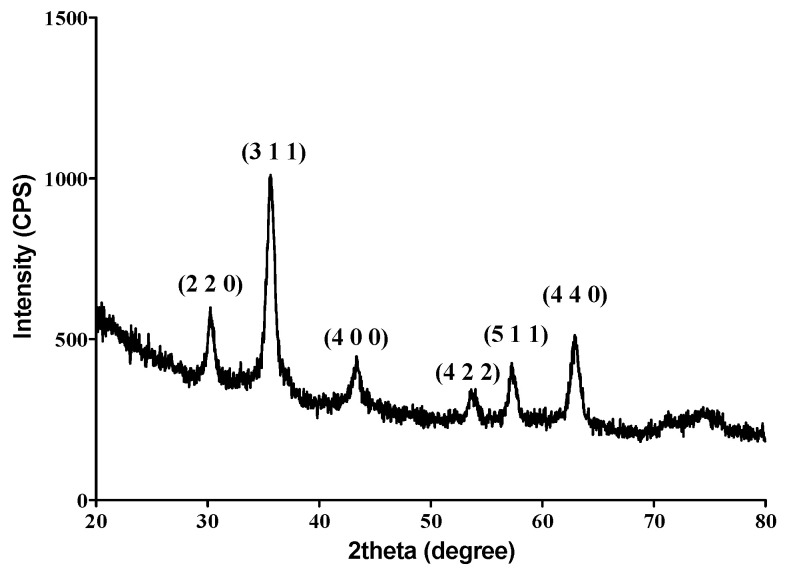
XRD pattern of Fe_3_O_4_@EG particles.

The XRD pattern (Figure 1) shows the presence of strong diffraction interferences that are characteristic for one mineralogical phase, namely magnetite (Fe_3_O_4_), as identified from the ICDD (International Centre of Diffraction Data) card No. 19-0629. Six specific diffraction planes corresponding to 2θ values of ~30, ~35, ~43, ~53, ~57 and ~62 (°) were thus identified, namely (2 2 0), (3 1 1), (4 0 0), (4 2 2), (5 1 1) and (4 4 0). The results are in concordance with previously reported data [83,84] on crystalline magnetite with a spinel cubic structure.

**Figure 2:**
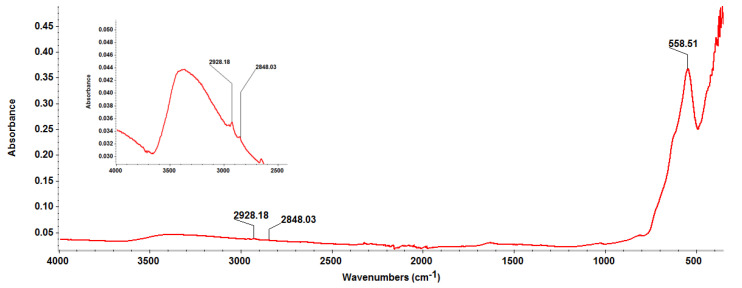
Fourier transform infrared spectroscopy (FT-IR) spectrum of Fe_3_O_4_@EG particles.

The IR spectrum from Figure 2 demonstrates the successful conjugation of EG molecules onto Fe_3_O_4_ particles. The vibrational bands identified at ~2928 cm^−1^ and ~2848 cm^−1^ correspond to the asymmetric and symmetric stretching of –CH_3_ originating from Eucalyptus essential oil [85], while the ~558 cm^−1^ band is assigned to Fe–O stretching vibrations from magnetite [86,87].

**Figure 3:**
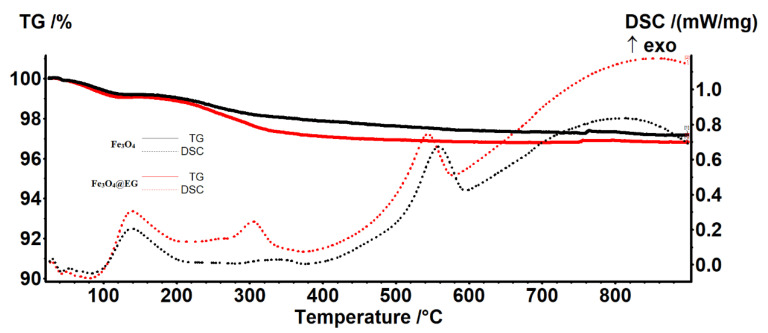
Thermal analysis of Fe_3_O_4_ and Fe_3_O_4_@EG particles.

To obtain qualitative and quantitative information on the composition of synthesized particles, comparative thermal studies (Figure 3) on pristine and EG-conjugated Fe_3_O_4_ were performed, using in this respect TGA and differential scanning calorimetry (DSC). The derivatogram corresponding to bare Fe_3_O_4_ evidences a total weight loss of 2.81%, which occurred in three steps: (i) the first weight loss, accompanied by an exothermic process, is recorded at 139.2 °C, and is attributed to the evaporation of water adsorbed on the surface of particles (water chemo-desorption); (ii) the second exothermic event (between 300–400 °C) has a lower amplitude, but is correlated with a reduction in the sample’s mass of 1.27%, which may be due to the elimination of absorbed water molecules as a result of the complete degradation of hydroxyl groups from the surface of iron oxide nanoparticles; and (iii) the last exothermic phenomenon comes together with a mass change of 0.73%, and may be attributed to the isomorphic transformation of magnetite into γ-maghemite [88,89].

In the case of Fe_3_O_4_@EG sample, a similar thermal behavior pattern was followed: there are three main exothermic events, accompanied by mass reductions. In comparison with the pristine iron oxide sample, the amplitude of the process that occurred at ~300 °C is much higher and comes together with an increased mass reduction (1.92%). This may result from the degradation of the organic molecules from Eucalyptus essential oil, which were conjugated onto the nanoparticles’ surface during the synthesis process. A total mass loss of 3.17% is noticed for the Fe_3_O_4_@EG sample. By considering both powdery samples’ thermal data, we can estimate the amount of EG essential oil as 0.36 ± 0.1 wt.%.

**Figure 4:**
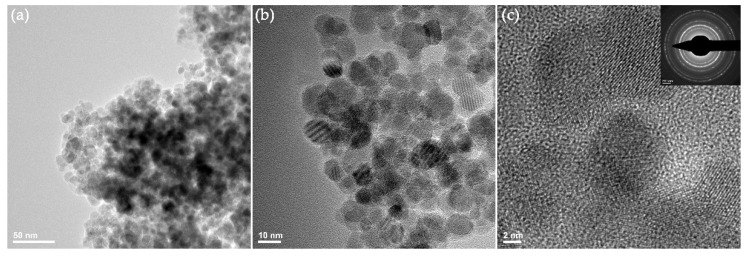
Transmission electron microscopy (TEM) (**a**,**b**) and HR-TEM (**c**) micrographs, and selected area electron diffraction (SAED) pattern (inset) of Fe_3_O_4_@EG particles.

Relevant microstructural aspects on the Fe_3_O_4_@EG particles were provided by transmission electron micrographs (Figure 4). A pronounced agglomeration tendency of the powdery sample is noticed (Figure 4a), together with the presence of individual nanoparticles that possess no preferential morphology and a non-homogenous aspect. A complementary micrograph (Figure 4b) indicates the quasi-spherical morphology of Fe_3_O_4_@EG particles and confirms their nanosize (average diameter of 7.5 ± 2.5 nm). It can also be noticed that the nanoparticles have a good dispersion in alcoholic suspension due to the stabilizing role provided by the phytochemical molecules contained in the EG essential oil. The high-resolution TEM micrograph (Figure 4c) emphasizes a preferential core/shell structure of the synthesized Fe_3_O_4_@EG systems, as inorganic nanoparticles of high crystallinity are individually dispersed in low crystalline organic mass. The SAED pattern of EG-conjugated nanoparticles (Figure 4c, inset) confirms the highly crystalline nature of obtained systems, while the identified diffraction planes enable us to categorize the sample as face-centered spinel structured magnetite. These results are in concordance with previously reported data [90,91], and with the above-discussed XRD results. Complementary results evidenced that Fe_3_O_4_@EG nanoparticles have hydrodynamic diameters between 200.9 and 247 nm, with negative surface charge (−1.7 mV zeta potential).

**Figure 5:**
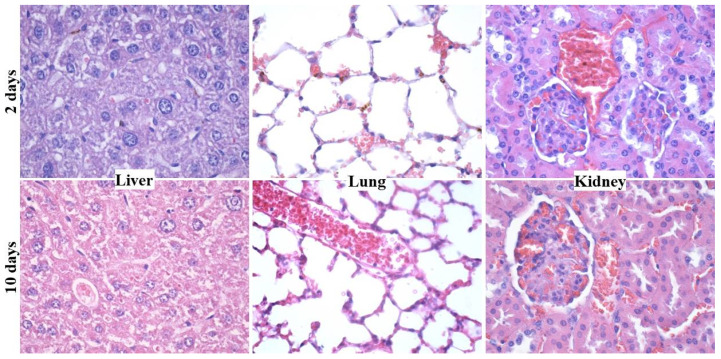
Optical micrographs of hepatic, pulmonary and renal tissues harvested after 2 and 10 days of treatment with Fe_3_O_4_@EG (400× magnification).

Preferential tissue retention of Fe_3_O_4_@EG particles was evidenced after systemic administration and histological evaluation. No morphological alterations, ultrastructural modifications or foreign systems were observed for the brain, myocardium and pancreas tissues, regardless of the treatment period. In comparison, dark-brown aggregates were noticed within capillaries and tissue-specific macrophages of liver and lung (hepatic stellate and pulmonary perivascular macrophages, respectively), at two days after inoculation. The presence of reduced-in-size aggregates was also noticed at the renal level, but only in the blood vessels, as they were absent in glomeruli, renal tubules and renal stroma. Still, no histological alterations were observed in neither of these tissues. After a prolonged treatment (10 days) with proposed nanosystems, the liver, lung and kidney were negative for the presence of Fe_3_O_4_@EG nanoparticles (no ultrastructural modifications or functional alterations were identified) (Figure 5).

**Figure 6:**
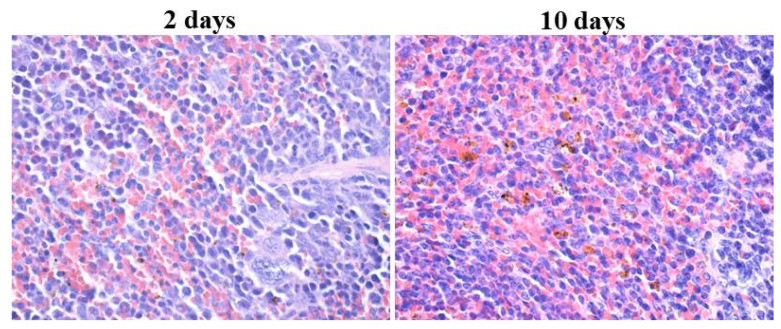
Optical micrographs of splenic tissue harvested after 2 and 10 days of treatment with Fe_3_O_4_@EG (400× magnification).

In the case of splenic tissue (Figure 6), nanoparticle-based aggregates were evidenced in the red pulp, with higher tissue concentration at 10 days after injection. Fe_3_O_4_@EG were absent in the white pulp of the spleen, regardless of the applied treatment. Instead, the splenic white pulp’s time-dependent hypertrophy was observed due to overstimulated production of multilobed nucleated macrophages, a process activated by the EO-conjugated magnetite nanoparticles.

The MAPLE technique gained a lot of attention for the development of biocompatible and biomimetic coatings for implantable materials and devices, thanks to its properties, such as controlled topography of resulted nanosized layers, strong adhesion between synthesized films and implant’s surface, and stoichiometric transfer of target materials onto the substrates [92]. Herein, we employed this versatile laser processing method to obtain composite PLA/Fe_3_O_4_@EG thin films at different laser fluences (200, 300 and 400 mJ/cm^2^).

To identify optimal parameters for MAPLE, the laser processed samples were compared with equivalent dropcast samples by IRM analysis, in terms of purity and stoichiometry (IR spectra), as well as structural integrity and efficient laser transfer (IR maps). For all specimens, IR spectra were recorded for different points on each sample, while the IR maps were built by monitoring the distribution of absorption bands from ~1750 cm^−1^ and ~2990 cm^−1^, corresponding to carbonyl (C=O) and methyl (–CH_3_) groups, respectively. Colors from each IR map are directly related the monitored absorption band’s intensity, ranging from blue to red (minimum to maximum absorbance intensity, respectively).

**Figure 7:**
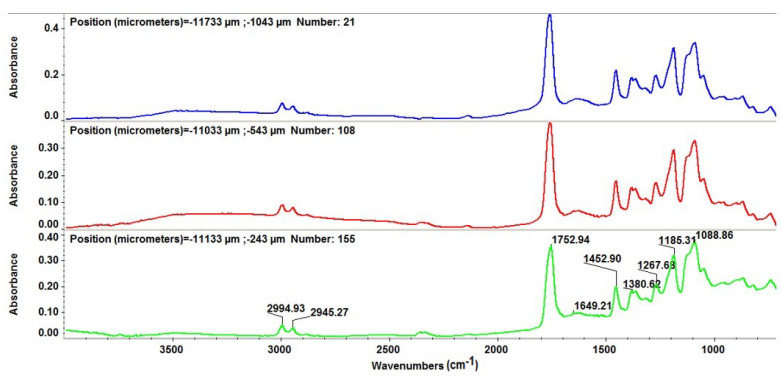
IR spectra of polylactic acid (PLA)/Fe_3_O_4_@EG dropcast.

The IR spectroscopy was employed in order to obtain important data on the chemical preservation and stoichiometric transfer of PLA/Fe_3_O_4_@EG material during the laser-assisted synthesis of thin films. For the dropcast sample, the IR spectrum (corresponding to the initial material) is depicted in Figure 7. Specific absorbance maxima in the composite sample are identified as corresponding to the following functional groups from both organic compounds: ~2995 cm^−1^ and ~2945 cm^−1^ (asymmetric and symmetric stretching of –CH_3_), ~1753 cm^−1^ (strong stretching of C=O), ~1453 cm^−1^ (overlapped bending of C–H and C=O) and ~1089 cm^−1^ (stretching of C–O) [93,94]. The IR peaks from ~1381 cm^−1^ and ~1185 cm^−1^ characterize bending of –CH_3_ and asymmetric stretching of C–O–C from PLA [95,96]. Stretching vibrations of C–O originating from the cyclic ether of eucalyptol may be assigned at ~1268 cm^−1^ [97,98].

**Figure 8:**
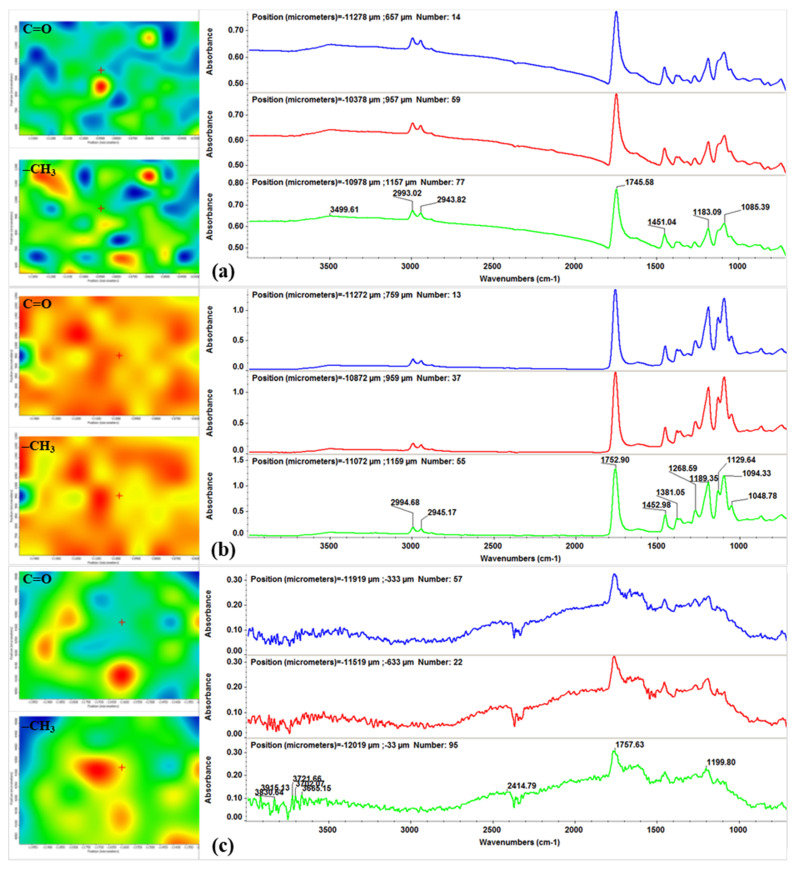
IR maps **(left**) and corresponding IR spectra (**right**) of PLA/Fe_3_O_4_@EG coatings processed by matrix-assisted pulsed laser evaporation (MAPLE) at 200 mJ/cm^2^ (**a**), 300 mJ/cm^2^ (**b**) and 400 mJ/cm^2^ (**c**).

By considering the color distribution from infrared maps (Figure 8, left) and its correlation with material’s transfer, one can notice that the value of laser fluence has a clear impact on the homogeneity of obtained coatings, which is directly related to functional groups repartition. The lowest distribution of PLA/Fe_3_O_4_@EG material onto the substrates (predominantly blue and green areas) is identified for the 200 mJ/cm^2^ laser fluence (Figure 8a, left), followed by the 400 mJ/cm^2^ laser fluence (Figure 8c, left). In the first case, this outcome may be related to an inefficient material transfer, as the corresponding IR spectra (Figure 8a, right) evidence the presence of less intense (or even absent) absorbance maxima when compared to the dropcast sample. For the highest laser fluence, the IR spectra (Figure 8c, right) point out significant degradation of previously identified functional groups. The most homogenous and uniform transfer of PLA/Fe_3_O_4_@EG material is attained by using the 300 mJ/cm^2^ laser fluence (Figure 8b, left), with no alterations in the chemical structure of composite material being observed (Figure 8b, right).

The infrared data confirm that composite coatings containing PLA and iron oxide nanoparticles conjugated with EG essential oil were successfully obtained. In terms of chemical integrity and efficient material transfer, the middle laser fluence (300 mJ/cm^2^) was experimentally identified as the optimal choice for the MAPLE synthesis of PLA/Fe_3_O_4_@EG coatings. Therefore, composite materials for further characterization (SEM investigation) and cellular evaluation (biological and microbiological assays) were obtained only by using this particular value of laser fluence.

**Figure 9:**
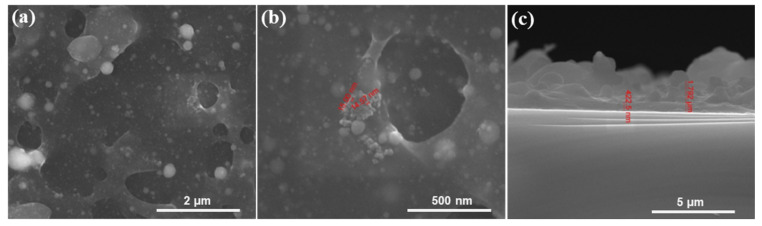
Plain view (**a**,**b**) and cross-section (**c**) scanning electron microscopy (SEM) micrographs of PLA/Fe_3_O_4_@EG coatings.

The surface morphology, structure and thickness of PLA/Fe_3_O_4_@EG samples obtained at 300 mJ/cm^2^ laser fluence were evaluated by the plain view and cross-section SEM analysis, respectively. The lower magnification image (Figure 9a) shows a uniform and complete coverage of the substrate by the composite material, with a particulate aspect (suggesting a highly irregular surface) and relatively homogenous aspect. The partial solubility of PLA in DMSO may be responsible for the formation of a continuous polymer layer (with predominant cavity-like structure) and the presence of spherical-shaped particulate structures (occurred due to intense reorganization of polymer following its interaction with the laser beam). At higher magnification (Figure 9b), one can notice that aggregates of Fe_3_O_4_@EG nanoparticles are embedded and uniformly distributed within the PLA matrix. The cross-section image from Figure 9c indicates a rough surface of PLA/Fe_3_O_4_@EG coating, which is a beneficial parameter for cellular interactions. The thickness of composite coating, with irregular surface morphology, ranges between 420 nm and 1.7 µm.

**Figure 10:**
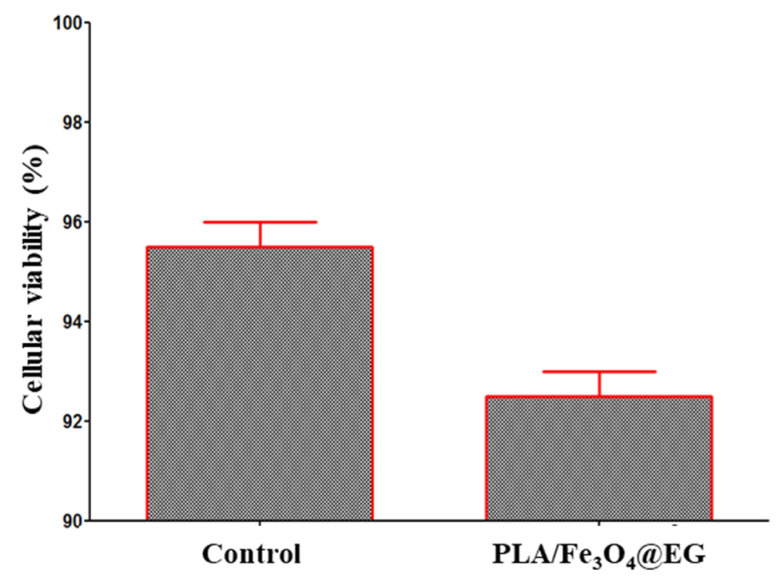
Viability results of human amniotic fluid-derived mesenchymal stem cells (AFSCs) incubated for 72 h in the presence of PLA/Fe_3_O_4_@EG coatings.

For the biological evaluation of PLA/Fe_3_O_4_@EG coatings, the percent of metabolically active stem cells incubated for 72 h in the presence of MAPLE processed samples was determined (Figure 10). The viability of AFSCs was calculated as reported to control cells (non-treated cells). The MTT assay results demonstrate that PLA/Fe_3_O_4_@EG coatings are favorable substrates for the normal development of AFSCs, since no modification in the metabolic activity of cells is evidenced after three days of treatment. In comparison with control specimens, a slightly decreased cellular viability (~3%) is noticed in the case of nanostructured composites, but the amount of viable cells is maintained above 92%. Besides their versatile composition and tunable characteristics (including mechanical and thermal response, permeability and solubility), the intrinsic biocompatibility and adjustable biodegradability of PLA-based biomaterials are essential aspects for the development of new and effective antimicrobial formulations [99,100,101,102]. The obtained biological results show that PLA/Fe_3_O_4_@EG coatings are highly biocompatible substrates for the normal growth and proliferation of human-derived cells and indicate them as suitable candidates for surface modification of mid-term implantable biomaterials and devices.

The ability of MAPLE processed nanostructured composite films to inhibit bacterial biofilm formation and maturation was evaluated at different time intervals against representative Gram-negative (*E. coli*) and Gram-positive (*S. aureus*) pathogens.

**Figure 11:**
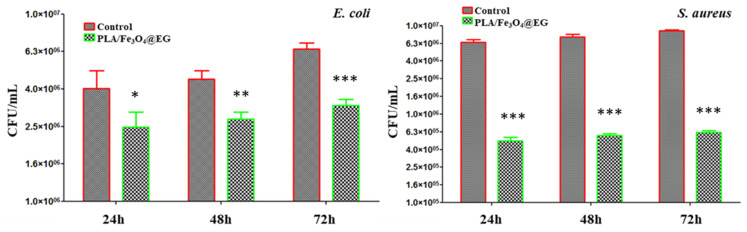
Microbial biofilm development of *E. coli* (**left**) and *S. aureus* (**right**) after different incubation periods with PLA/Fe_3_O_4_@EG coatings, expressed as CFU/mL values reflecting number of viable cells imbedded in biofilms. * *p* < 0.05; ** *p* < 0.01; *** *p* < 0.001 (CFU/mL values of control vs. PLA/Fe_3_O_4_@EG coatings).

In the case of *E. coli* strain (Figure 11, left), PLA/Fe_3_O_4_@EG coatings determine the decrease of the bacterial population by ~2.5 folds, both after 24 h and 48 h of treatment. It can be clearly noticed that after 72 h of incubation in the presence of composite materials, the CFU/mL values are reduced by three times when compared to control values. Regarding the impact of PLA/Fe_3_O_4_@EG coatings on *S. aureus* biofilm development (Figure 11, right), the inhibition level is more evident, since bacterial populations are reduced by at least 10 folds, regardless of the incubation time. These overall results evidence the effectiveness of PLA/Fe_3_O_4_@EG composite films as anti-biofilm surfaces.

As a general remark, the inhibition of biofilm development by PLA/Fe_3_O_4_@EG coatings is comparable for all investigated time intervals for both bacteria. Such behavior indicates the sustained efficiency of proposed nanostructured materials against microbial biofilm development, as they proved to interfere with the early stage formation of biofilms (contamination and colonization), but also with the maturation phase. The particular anti-biofilm effect evidenced against *S. aureus* relies on the compositional and microstructural differences between the cells of selected pathogens [103,104]. Although the exact antimicrobial mechanism is not known, we assume synergistic effects due to EO and magnetite nanoparticles, both with proved antimicrobial activity. The main antimicrobial mechanism of *Eucalyptus globulus* EO is the membrane cell damage and interference with proton pumps and electrolyte channels [105,106]. On the other hand, magnetite nanoparticles are known for their cell wall damage properties, as well as stimulation of the intracellular release of reactive oxygen species (ROS), which interfere with vital mechanisms, such as cell division and signaling [107]. Improved antimicrobial activity of synthetic [108,109] and natural [110,111] antibiotics were reported when conjugated with magnetite nanoparticles.

Herein, the biological results evidence that PLA/Fe_3_O_4_@EG coatings are highly biocompatible substrates for human-derived cells. The microbiological data indicate that nanostructured PLA/Fe_3_O_4_@EG materials are suitable candidates for anti-biofilm surface modification of short-term to mid-term implantable biomaterials and devices.

## 4. Conclusions

The present study reports the successful synthesis of PLA/Fe_3_O_4_@EG nanocomposite coatings and their potential use as biocompatible and anti-biofilm surfaces for implantable biomaterials and devices. By using a modified co-precipitation protocol, ultra-small (7.5 ± 2.5 nm) highly crystalline magnetite nanoparticles were conjugated with a natural antibiotic, Eucalyptus essential oil. The MAPLE technique provided an efficient, uniform and stoichiometric synthesis of nanostructured composite thin films. The cellular assays proved that the PLA/Fe_3_O_4_@EG coatings did not affect the viability and proliferation of eukaryote cells, while significantly interfered with the formation and maturation of bacterial biofilms. Thus, the proposed PLA/Fe_3_O_4_@EG nanocomposite coatings represent suitable candidates for surface modification of implantable biomaterials and devices by enhancing their biocompatibility and inducing or potentiating their anti-infective effects.

## Data Availability

The data presented in this study are available on request from the corresponding author.
